# Automated assessment of collateral circulation and infarct core: predictors of functional outcomes in acute ischemic stroke following endovascular thrombectomy

**DOI:** 10.1007/s00234-024-03519-4

**Published:** 2025-02-04

**Authors:** Ingrid Požar, Fajko F. Bajrović, Lan Umek, Katarina Šurlan Popović

**Affiliations:** 1 Department of Radiology, Izola General Hospital, Izola, Slovenia; 2 Faculty of Medicine, University of Ljubljana, Ljubljana, Slovenia; 3 Department of Vascular Neurology and Intensive Care, University Medical Centre Ljubljana, Ljubljana, Slovenia; 4 Institute of Pathophysiology, Faculty of Medicine, University of Ljubljana, Ljubljana, Slovenia; 5 Faculty of Public Administration, University of Ljubljana, Ljubljana, Slovenia; 6 Institute of Radiology, University Medical Centre Ljubljana, Ljubljana, Slovenia

**Keywords:** Collateral circulation, Infarct core, Acute ischemic stroke, CT angiography, CT perfusion

## Abstract

**Purpose:**

This study aimed to evaluate the predictive value of automatically assessed collateral circulation (CC) and infarct core for functional outcome in acute ischemic stroke (AIS) patients treated with endovascular thrombectomy (EVT).

**Methods:**

We conducted a retrospective cohort study of 208 patients with anterior large vessel occlusion treated with EVT. Two AI-powered software were used to automatically assess CC and infarct core. Comparative analyses included patient demographics, clinical and imaging data, and functional outcome. Univariate and multivariable logistic regression analyses were conducted to predict the 90-day functional outcome. A favorable outcome was defined as a modified Rankin scale (mRS) score ≤ 2.

**Results:**

Among the 208 patients, 114 (54.8%) were women and 94 were men, with a mean age of 71.4 ± 13.3 years. Patients with higher collateral score (CS) exhibited lower infarct core volumes (*p* < 0.001) and better mRS score at 90 days (*p* = 0.008). Among patients with a favorable outcome, the mean infarct core volume was lower compared to those with poor outcomes (5 mL vs. 8.6 mL, *p* = 0.003). In univariate logistic regression, both infarct core (OR 0.94, *p* = 0.005) and CS (OR 1.84, *p* = 0.014) were predictors of favorable outcome. However, in multivariable models, only infarct core remained a significant independent predictor [AORs of 0.95 (*p* = 0.021) and 0.96 (*p* = 0.039)].

**Conclusion:**

Automatically assessed infarct core is a robust predictor of functional outcome in AIS patients post-EVT, while CS’s predictive value diminishes when adjusted for infarct core. These findings support the integration of AI-powered evaluations in clinical settings to improve prognosis and treatment strategies for AIS.

## Introduction

Acute ischemic stroke (AIS) is a worldwide problem, causing a high number of deaths and disability on a global scale [1]. The primary goals of acute stroke therapy are to restore blood flow to the ischemic but salvageable tissue, known as the penumbra, and to minimize the expansion of the irreversibly damaged area, referred to as the infarct core. Endovascular treatment for AIS has demonstrated significant effectiveness in reducing both disability and mortality rates among patients [2].

Numerous studies have highlighted the importance not only of timely reperfusion but also of cerebral collateral circulation (CC) in stroke outcomes, with the role of CC and its extent remaining not yet fully understood [3–5]. CC sustains viability in hypoperfused tissue and mitigates ischemic injury [6]. Consequently, good CC is associated with smaller infarct cores and favorable functional outcomes [7, 8]. Furthermore, recent research underscores robust CC as a significant predictor of good clinical outcomes [9].

Multiple CC grading methods are currently utilized, resulting in challenges when comparing studies and applying them clinically. These methods primarily rely on visual assessments conducted by experts (i.e. visual scoring), thereby introducing subjectivity. To address this, various artificial intelligence (AI)-powered software solutions have emerged, producing automated collateral score (CS) values to uniformize and deindividualize grading systems. Utilizing AI-driven software offers ease of use, rapid processing, and independence from operator influence, thereby enhancing and standardizing CC evaluations [4, 10].

Beyond CC, the correlation between infarct core size and patient outcome has also been extensively described, with smaller infarct cores associated with favorable outcomes and serving as a strong predictor of clinical outcome [11, 12]. Due to their known correlation with patient outcomes in AIS, significant focus has been placed on the combined roles of CC and infarct core [13]. Recent literature describes their combined effect in predicting poor outcomes, although these studies rely on visual scoring of CC [14]. However, there is a lack of data on their combined predictive value when both CC and infarct core are obtained automatically using AI-powered software.

The purpose of this study was to evaluate the predictive value of automatically assessed CC and infarct core for functional outcome in AIS patients treated with endovascular thrombectomy (EVT).

## Methods

### Patient selection

In this retrospective cohort study, we included 208 consecutive patients with AIS in the anterior cerebral circulation who were treated with EVT at the Neurology Clinic of the University Medical Centre Ljubljana, Slovenia, between June 2018 and November 2020. The study was approved by the National Medical Ethics Committee of the Republic of Slovenia (No. 0120–377/2019/4). All procedures performed were part of routine care. Informed consent was obtained for all individual participants included in the study, either from the participants themselves or, where applicable, from their relatives.

Study inclusion criteria were as follows: (1) age ≥ 18 years, (2) large vessel occlusion in the anterior cerebral circulation confirmed on CT angiography (CTA), (3) perfusion deficit in the anterior cerebral circulation confirmed on CT perfusion (CTP), (4) treatment with EVT, and (5) clinical follow-up at 3 months. Patients were excluded if they met any of the following criteria: (1) previous ischemic stroke lesion ≥ 2.5 cm, (2) encephalomalacia lesion ≥ 2.5 cm, (3) absence of a suitable CT protocol, or (4) presence of extreme artifacts affecting accurate image analysis. In our study, we included all patients eligible for the EVT procedure, including those with early and late symptom onset, as well as those with an unknown time of symptom onset.

### Study design

The study followed a retrospective design. All included patients underwent an initial comprehensive multimodal CT evaluation during the emergency assessment for AIS (baseline CT), followed by a non-contrast CT (NCCT) 24 h after EVT (follow-up CT). All CT scans performed were part of routine clinical care, following the established protocol for managing AIS patients. After the baseline CT evaluation, all patients underwent EVT treatment. The procedure was performed by an experienced interventional neuroradiologist, who assessed the degree of artery recanalization upon completion. Additionally, each patient was examined by an expert neurologist during their urgent care and again 90 days post-discharge. Functional status assessments, based on the modified Rankin Scale (mRS), were conducted as part of the standard protocol at the Neurology Clinic of the University Medical Centre Ljubljana.

A comprehensive review of retrospective data was conducted for all patients, including demographic characteristics, mortality data, time from stroke onset or last known well to baseline CT, time from stroke onset or last known well to EVT, etiology of acute ischemic stroke, IVT therapy, disability data, and imaging data. Imaging data included CS score, infarct core volume, site of vessel occlusion, recanalization status, occurrence of early ischemic changes, ischemic lesion demarcation, and hemorrhagic transformation. Patient disability status was evaluated using the mRS scale, with three assessments: the previous mRS score (pre-stroke), the baseline mRS score at admission to the emergency department, and the mRS score at 90 days at follow-up. A favorable functional outcome at the 90-day follow-up was defined as an mRS score of ≤ 2.

### Imaging protocol

CT examinations were performed at the Department of Neuroradiology at the Neurology Clinic in Ljubljana, using a Siemens Somatom Sensation Open scanner (Siemens, Erlangen, Germany). The baseline work-up included NCCT, single-phase CTA, and CTP. A follow-up NCCT was conducted 24 h after EVT. All CT scans performed were part of standard care.

All NCCTs were acquired in axial mode from the skull base to the vertex (16 cm z-axis coverage) using the following imaging parameters: (1) Skull base: 120 kV peak tube voltage, 265 mAs, slice thickness of 3 mm, 1 s rotation time, 32 cm scan field of view (SFOV), and a 512 × 512 matrix; and (2) Head: 120 kV peak tube voltage, 310 mAs, slice thickness of 4.8 mm, 1 s rotation time, 32 cm SFOV, and a 512 × 512 matrix.

CTA in helical mode was performed from the aortic arch to the vertex using the following imaging parameters: 120 kV peak tube voltage, 150–200 mAs, slice thickness of 0.6 mm, rotation time of 0.33–0.5 s, and a pitch of 1.2–1.4. A power injector was used to administer 80 mL of contrast agent at a flow rate of 4 mL/s. The delay was set using CareBolus, with a region of interest (ROI) in the ascending aorta and a trigger threshold value of 100 Hounsfield units (HU).

All CTP series were acquired in axial scan mode using the following imaging parameters: 80 kV peak tube voltage, 210 mAs, 32 cm SFOV, and a 512 × 512 matrix. CTP images were obtained at the level of the basal ganglia and the third ventricle, covering a z-axis of 60 mm, with a slice thickness of 5 mm. The CTP images were acquired for 40 s, with a 2-second delay after the start of the injection of 40 mL of contrast agent at a flow rate of 6 mL/s using a power injector.

All patients received a tri-iodinated non-ionic monomeric low-osmolality contrast agent (Iomeron^®^ 400 mg/mL, Bracco, Milan, Italy).

### Image analysis

**Fig. 1:**
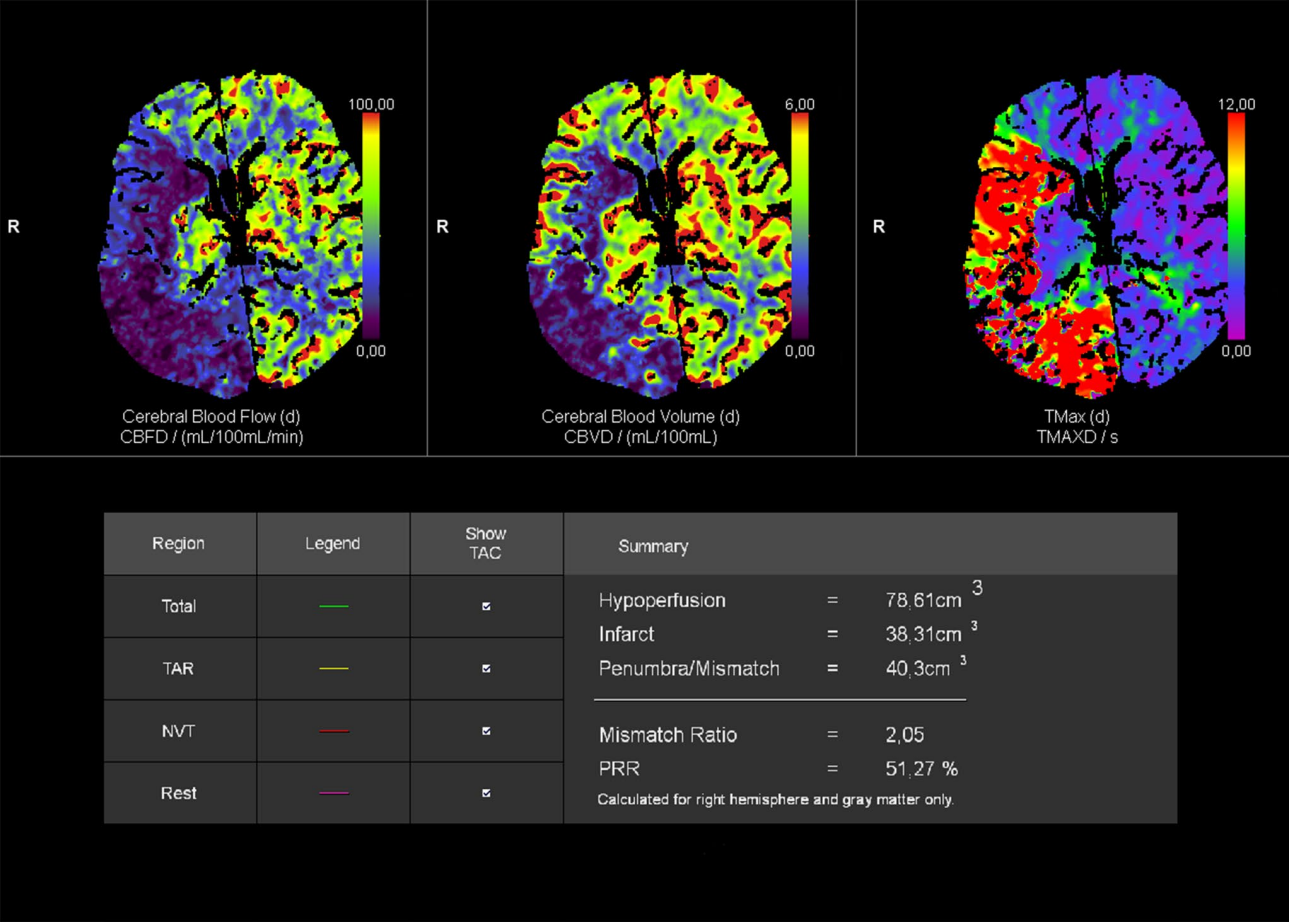
Representative case of a CT perfusion scan analyzed with syngo.CT Neuro Perfusion software. From left to right, the different color-coded perfusion maps depict cerebral blood flow, cerebral blood volume, and Tmax (time to maximum of the residue function), respectively, showing abnormal perfusion in the affected right brain hemisphere. Below, the table summarizes automatically calculated perfusion volumes and ratios, including the infarct core volume (38.31 cm³)

**Fig. 2:**
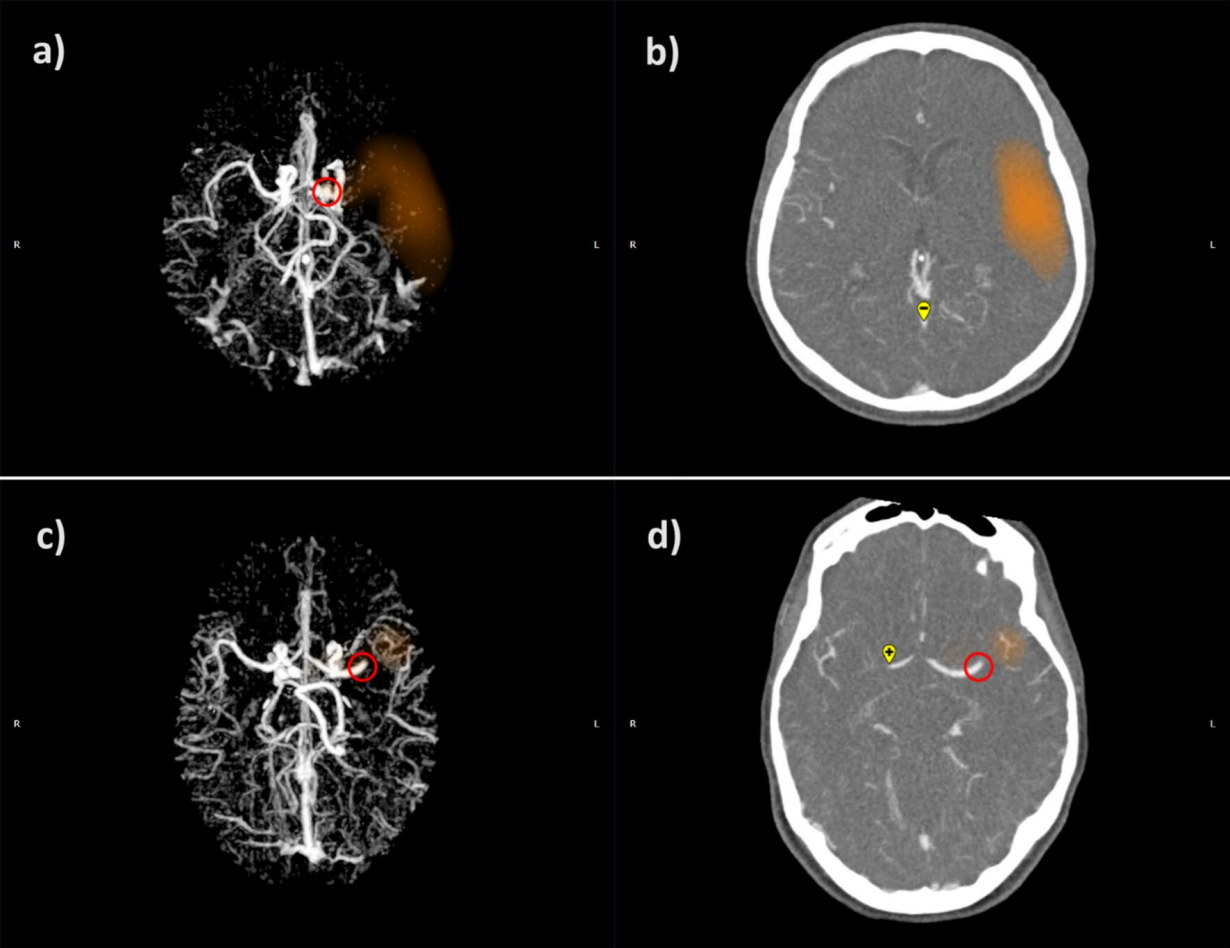
Representative cases of two distinct CTA scans analyzed with Brainomix’s software, illustrating patients with different collateral statuses: (**a**) and (**b**) show a patient with fair collaterals (CTA-CS 1), while (**c**) and (**d**) depict a patient with good collaterals (CTA-CS 3). Axial CTA MIP reconstructions are shown in (**a**) and (**c**), and axial CTA scans are shown in (**b**) and (**d**). The red circle indicates the site of vessel occlusion, and the orange area represents the location and extent of the affected territory within the MCA vascular region. The plus and minus signs represent the artery inflow and venous outflow measuring points, respectively

Imaging assessments were conducted using two AI-driven, automated, and validated software packages. The syngo.CT Neuro Perfusion software (Siemens Healthineers AG, Forchheim, Germany) was used to evaluate the infarct core volume, which was automatically calculated in cm³ (equivalent to mL). The software employs a standardized threshold to delineate the infarct core, defined as a relative cerebral blood flow (rCBF) value of less than 30% of normally perfused brain tissue (rCBF < 30%). Representative CTP images are shown in Fig. 1. The e-STROKE SUITE software (Brainomix Ltd., Oxford, UK) was utilized to automatically generate the CS and ASPECTS (Alberta Stroke Program Early CT Score) scores, as well as to determine the site of large vessel occlusion. The CS was automatically generated by the software, which utilizes a machine learning algorithm for fully automated calculation. Collateral status was evaluated and expressed by the software using the predefined CTA-CS (Computed Tomography Angiography Collateral Score) score, ranging from 0 to 3. This scoring system is integrated within the software and is based on the grading method proposed by Tan et al. [15]. The CTA-CS score represents the percentage of the affected middle cerebral artery territory filled by collateral supply compared to the contralateral, unaffected cerebral hemisphere: score 0 indicates less than 10% filling, score 1 indicates 10–50%, score 2 indicates 51–90%, and score 3 indicates more than 90% filling. Thus, a higher CTA-CS score indicates better collateral circulation. In our study, patients were categorized into four groups based on their CTA-CS scores. For the purposes of our research, we also dichotomized the CTA-CS scale: we defined good collaterals as a CTA-CS score of 3, while fair collaterals were defined as a CTA-CS score of 0–2, similar to previous studies [16]. Representative CTA images of good and fair collaterals as depicted by e-STROKE SUITE are shown in Fig. 2. Furthermore, the ASPECTS score was utilized to assess the presence and extent of early ischemic changes. Finally, the site of large vessel occlusion was identified as either the internal carotid artery (ICA), the middle cerebral artery (MCA), or the anterior cerebral artery (ACA). For image analysis, we used the vendor-specified default settings and thresholds for both software packages.

On the follow-up NCCT scan at 24 h after EVT, an expert neuroradiologist assessed the presence of possible hemorrhagic transformation or ischemic lesion demarcation.

The degree of vessel recanalization was evaluated by an expert interventional neuroradiologist at the end of the EVT procedure using the modified thrombolysis in cerebral infarction (mTICI) score. Successful reperfusion was defined as an mTICI score of ≥ 2b.

### Statistical analyses

The Kolmogorov-Smirnov test was employed to assess the normality of data distribution. Given the significant deviation from normality observed across all numerical variables, we opted for non-parametric tests for analysis. Despite this, for better clarity and to account for the absence of extreme outliers, means and standard deviations (SD) were reported instead of medians and interquartile ranges (IQR).

We classified the patients into four categories based on their CTA-CS scores (0–3) and conducted a bivariate analysis to investigate disparities between these categories, using the Kruskal-Wallis H test for continuous variables and Pearson’s chi-square test for categorical variables.

To assess the functional outcome at 90 days, we dichotomized the subjects into two groups: favorable outcome (mRS 0–2) and poor outcome (mRS 3–6). We then performed a bivariate analysis of these groups to investigate disparities, using the Mann-Whitney U test for continuous variables and Pearson’s chi-square test or Fisher’s exact test for categorical variables.

Both variables, CS and infarct core, were subsequently incorporated into univariate and multivariable logistic regression models to predict the functional outcome at 90 days. The variables included in the modeling met the inclusion criterion of achieving statistically significant *p*-values.

Lastly, we analyzed the distribution of infarct core volume values based on patient survival status using the Mann-Whitney U test.

For the statistical analyses, we used the SPSS software v.29 (IBM Corp., New York, USA) and the Python extension libraries Pandas and Matplotlib. Statistical significance for all analyses was defined as a *p*-value ≤ 0.05.

## Results

### Patient characteristics

A total of 208 patients met the inclusion criteria and were evaluated. Among them, 114 (54.8%) were women and 94 (45.2%) were men, with a mean age of 71.4 ± 13.3 years. IVT therapy was administered to 32.9% of the patients. The mortality rate in our patient population was 13% (*n* = 27). The median baseline mRS score at presentation was 5 (IQR 5–5). The etiology of AIS was attributed to atrial fibrillation in 54.3% of cases, carotid artery disease in 19.2%, and arterial dissection in 7.2%, while the etiology was unknown in the remaining 19.3%. The most common site of arterial occlusion was the MCA (95.7%), followed by the ICA (13.9%) and the ACA (2.4%). The characteristics of our patient population are summarized in Table 1.

**Table 1:** Baseline characteristics of CTA-CS categories

	All patients (*n* = 208)	CTA-CS 1 (*n* = 10)	CTA-CS 2 (*n* = 72)	CTA-CS 3 (*n* = 126)	*p*-value
Age, mean (SD) [years]	71.4 (13.3)	77.6 (6.9)	73.3 (12.1)	69.8 (14.1)	0.068
Sex (women), n (%)	114 (54.8)	4 (40)	35 (48.6)	75 (59.5)	0.209
IVT^*^, n (%)	68 (32.9)	2 (20)	27 (37.5)	39 (31.2)	0.447
ASPECTS^**^, median (IQR)	9 (8–10)	6 (4–9)	8 (7–9)	9 (8–10)	*< 0.001*
Infarct core, mean (SD) [mL]	6.8 (8.7)	25.5 (14.3)	8.9 (8.5)	4.2 (5.7)	*< 0.001*
Hemorrhagic transformation, n (%)	26 (12.5)	1 (10)	10 (13.9)	15 (11.9)	0.894
Ischemic lesion demarcation, n (%)	175 (84.1)	10 (100)	61 (84.7)	104 (82.5)	0.342
Mortality, n (%)	27 (13)	4 (40)	10 (13.9)	13 (10.3)	*0.026*
Previous mRS^*^, median (IQR)	0 (0–0)	0 (0–0)	0 (0–0)	0 (0–0)	0.366
Baseline mRS, median (IQR)	5 (5–5)	5 (5–5)	5 (5–5)	5 (4–5)	0.053
mRS at 90 days, median (IQR)	3 (1–4)	4 (3–6)	3 (1–4)	2 (1–4)	*0.008*
mTICI score^***^, median (IQR)	3 (2b-3)	2b (2b-2b)	3 (3–3)	3 (2b-3)	*0.003*
Time to CT^****^, mean (SD) [min]	164 (177)	193 (322)	147 (163)	172 (169)	0.279
ICA occlusion, n (%)	29 (13.9)	0 (0)	14 (19.4)	15 (11.9)	0.144
MCA occlusion, n (%)	199 (95.7)	10 (100)	69 (95.8)	120 (95.2)	0.773
ACA occlusion, n (%)	5 (2.4)	0 (0)	3 (4.2)	2 (1.6)	0.459
Atrial fibrillation, n (%)	113 (54.3)	8 (80)	42 (58.3)	63 (50)	0.130
Carotid artery disease, n (%)	40 (19.2)	0 (0)	8 (11.1)	32 (25.4)	*0.014*
Arterial dissection, n (%)	15 (7.2)	1 (10)	6 (8.3)	8 (6.4)	0.822

Missing data for ^*^1, ^**^5, ^***^13, and ^****^21 patients

CTA-CS (Computed tomography angiography collateral score: 0, < 10% of the affected middle cerebral artery territory filled by collateral supply compared to the contralateral unaffected hemisphere; 1, 10–50%; 2, 51–90%; 3, > 90%. There were no patients in the CTA-CS 0 category), IVT (intravenous thrombolysis), ASPECTS (Alberta stroke program early CT score), mRS (modified Rankin scale), mTICI (modified thrombolysis in cerebral infarction), time to CT (time from stoke onset or last known well to baseline CT), ICA (internal carotid artery), MCA (middle cerebral artery), ACA (anterior cerebral artery). Statistically significant *p*-values are italicized

### Collateral status

**Fig. 3:**
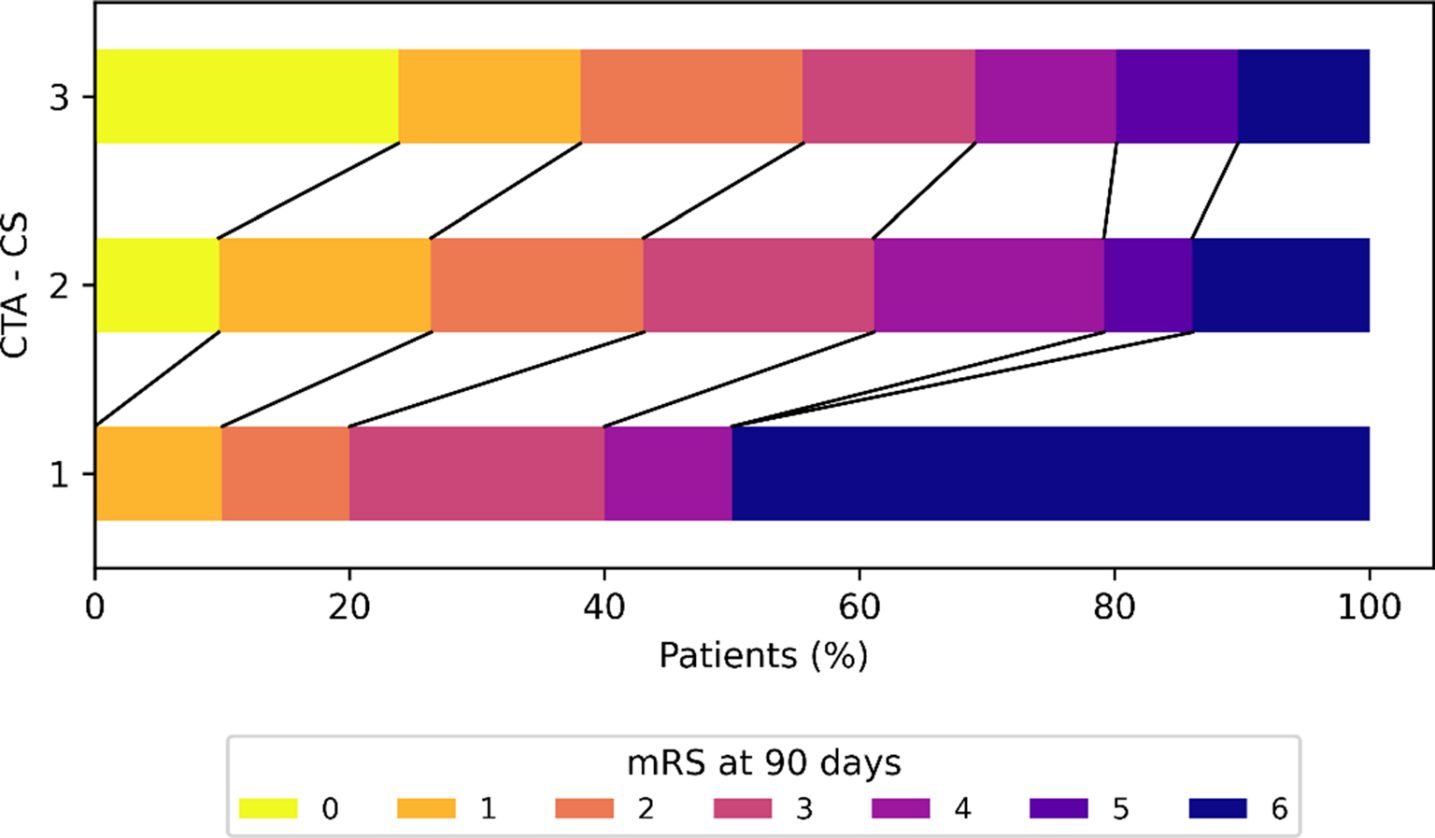
Distribution of mRS score at 90 days according to the CTA-CS (Computed Tomography Angiography Collateral Score) score. The CTA-CS score ranges from 0 to 3 based on the percentage of the affected middle cerebral artery territory filled by collateral supply compared to the contralateral unaffected hemisphere: 0 (< 10%), 1 (10–50%), 2 (51–90%), and 3 (> 90%). In the CTA-CS 0 category, no patients were observed. The mRS score, representing the modified Rankin scale, spans from 0 (no symptoms) to 6 (death)

Patients were classified into four CTA-CS categories based on their CS, as follows: there were no patients in the CTA-CS 0 category (0%), 10 patients in the CTA-CS 1 category (4.8%), 72 patients in the CTA-CS 2 category (34.6%), and 126 patients in the CTA-CS 3 category (60.6%). The results of the bivariate analysis of these categories are shown in Table 1. Patients with a higher CS displayed several significant differences compared to those with a lower CS, including a higher ASPECTS score, lower mean infarct core volume, lower mortality rate, higher prevalence of carotid artery disease, and higher mTICI score. It’s worth noting that patients in the CTA-CS 2 category had a non-significantly higher mTICI score compared to those in the CTA-CS 3 category (*p* = 0.679). Additionally, patients with a higher CS had a better mRS score at the 90-day follow-up. The median mRS at 90 days was 4 (IQR 3–6) in the CTA-CS 1 category, 3 (IQR 1–4) in the CTA-CS 2 category, and 2 (IQR 1–4) in the CTA-CS 3 category (*p* = 0.008). The distribution of mRS score at 90 days according to the CTA-CS category is shown in Fig. 3.

Furthermore, no statistically significant differences were observed between the CTA-CS categories in the variables sex, age, time to CT, IVT therapy, hemorrhagic transformation, ischemic lesion demarcation, previous mRS score, baseline mRS score, other stroke etiologies, and site of arterial occlusion.

### Functional outcome

**Fig. 4:**
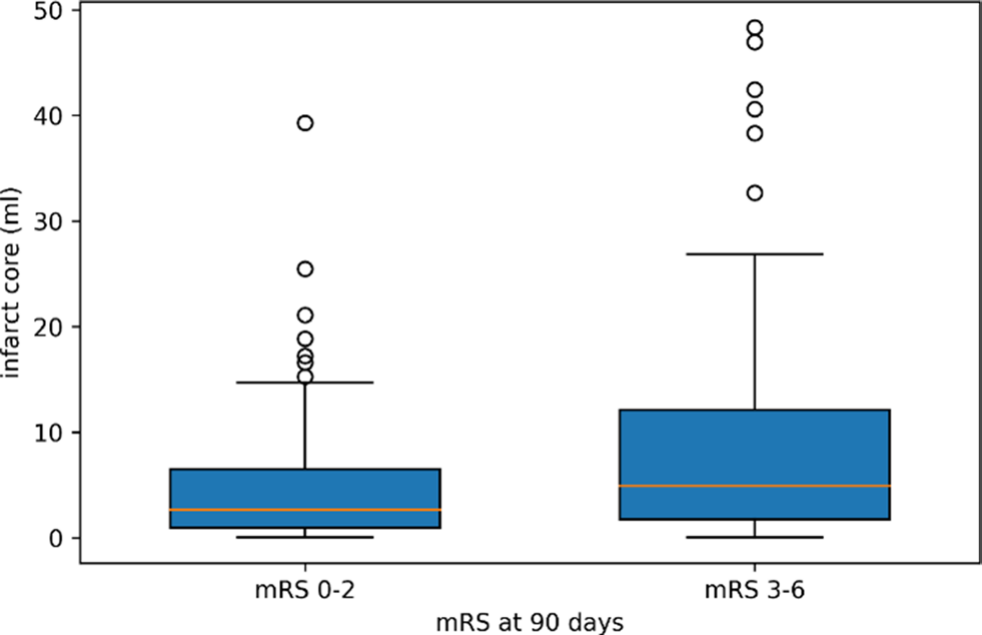
Distribution of infarct core volumes based on functional outcome, assessed with the mRS (modified Rankin scale) score at 90 days. We compared favorable (mRS 0–2) with poor (mRS 3–6) outcomes

To evaluate functional outcomes at 90 days, subjects were categorized into two groups: favorable (mRS 0–2) and poor (mRS 3–6) outcomes. The bivariate analysis results for these groups are detailed in Table 2. Of all patients, 103 (49.5%) had a favorable outcome, while 105 (50.5%) had a poor outcome. Patients with a favorable outcome were significantly younger, had higher ASPECTS and mTICI scores, lower previous mRS and baseline mRS scores, and exhibited less hemorrhagic transformation and ischemic lesion demarcation on follow-up CT scans. Additionally, a higher CTA-CS category was associated with better functional outcomes (*p* = 0.019), with 68% of patients in the favorable outcome group having good collaterals compared to 53.3% in the poor outcome group (*p* = 0.031). Furthermore, among patients with a favorable outcome, the mean infarct core volume was lower compared to the poor outcome group (5 mL vs. 8.6 mL, *p* = 0.003), as depicted in Fig. 4. No statistically significant differences were found between the two outcome groups in the variables sex, time to CT, time to EVT, successful EVT, IVT therapy, site of arterial occlusion, and stroke etiology.

**Table 2:** Bivariate analysis of functional outcome at 90 days

	Favorable outcome, mRS 0–2 (*n* = 103)	Poor outcome, mRS 3–6 (*n* = 105)	*p*-value
Age, mean (SD) [years]	66.9 (14)	75.7 (11.1)	*< 0.001*
Sex (women), n (%)	53 (51.5)	61 (58.1)	0.336
IVT^*^, n (%)	35 (34)	33 (31.7)	0.730
Previous mRS^*^, median (IQR)	0 (0–0)	0 (0–0)	*0.050*
Baseline mRS, median (IQR)	5 (4–5)	5 (5–5)	*< 0.001*
ASPECTS^**^, median (IQR)	9 (8–10)	8 (7–9)	*0.001*
CTA-CS category, n (%)			
0	0 (0)	0 (0)	*0.019*
1	2 (1.9)	8 (7.6)	
2	31 (30.1)	41 (39.1)	
3	70 (68)	56 (53.3)	
Good collaterals (vs. fair), n (%)	70 (68)	56 (53.3)	*0.031*
Infarct core, mean (SD) [mL]	5 (6.2)	8.6 (10.3)	*0.003*
Hemorrhagic transformation, n (%)	6 (5.8)	20 (19)	*0.004*
Ischemic lesion demarcation, n (%)	80 (77.7)	95 (90.5)	*0.011*
mTICI score^***^, median (IQR)	3 (3–3)	3 (2b-3)	*0.002*
Successful EVT (vs. unsuccessful) ^***^, n (%)	97 (97)	86 (90.5)	0.060
Time to CT^****^, mean (SD) [min]	171.2 (208.1)	157.4 (141.4)	0.607
Time to EVT^*****^, mean (SD) [min]	234.4 (148.6)	227.6 (104.7)	0.568
ICA occlusion, n (%)	11 (10.7)	18 (17.1)	0.178
MCA occlusion, n (%)	100 (97.1)	99 (94.3)	0.498
ACA occlusion, n (%)	2 (1.9)	3 (2.9)	1.000
Atrial fibrillation, n (%)	58 (56.3)	55 (52.4)	0.569
Carotid artery disease, n (%)	20 (19.4)	20 (19)	0.946
Arterial dissection, n (%)	10 (9.7)	5 (4.8)	0.168

Missing data for ^*^1, ^**^5, ^***^13, ^****^21, and ^*****^23 patients

mRS (modified Rankin scale), favorable outcome (mRS 0–2), poor outcome (mRS 3–6), IVT (intravenous thrombolysis), ASPECTS (Alberta stroke program early CT score), CTA-CS (Computed tomography angiography collateral score: 0, < 10% of the affected middle cerebral artery territory filled by collateral supply compared to the contralateral unaffected hemisphere; 1, 10–50%; 2, 51–90%; 3, > 90%), good collaterals (CTA-CS 3), fair collaterals (CTA-CS 0–2), mTICI (modified thrombolysis in cerebral infarction), successful EVT (mTICI 2b-3), unsuccessful EVT (mTICI 0-2a), time to CT (time from stoke onset or last known well to baseline CT), time to EVT (time from stoke onset or last known well to endovascular thrombectomy), ICA (internal carotid artery), MCA (middle cerebral artery), ACA (anterior cerebral artery). Statistically significant *p*-values are italicized

### Univariate and multivariable analyses of functional outcome

Results of univariate and multivariable logistic regression analyses to predict the favorable functional outcome (mRS 0–2) at 90 days are presented in Table 3. In the univariate model, infarct core was inversely associated with a favorable outcome (OR 0.94, 95% CI [0.91–0.98], *p* = 0.005), while patients with higher CS had 1.84 times greater odds of a favorable outcome (95% CI [1.13–2.99], *p* = 0.014) compared to those with lower CS. However, in the multivariable logistic regression analyses, CS lost significance, while infarct core emerged as an independent predictor. After adjusting the odds ratio, infarct core remained inversely associated with a favorable outcome, with adjusted odds ratios (AORs) similar to those of the univariate model (AORs 0.95 and 0.96).

**Table 3:** Univariate and multivariable logistic regression analyses for favorable outcome at 90 days

**Univariate models**	**OR**	**95% CI**	***p*** **-value**
Model 1			
Infarct core (1 mL increase)	0.94	0.91–0.98	0.005
Model 2			
CTA-CS	1.84	1.13–2.99	0.014
**Multivariable models**	**AOR**	**95% CI**	***p*** **-value**
Model 3			
Infarct core (1 mL increase)	0.96	0.92–0.99	0.039
CTA-CS	1.39	0.79–2.42	0.249
Model 4			
Infarct core (1 mL increase)	0.95	0.91–0.99	0.021
Good collaterals (vs. fair)	1.18	0.87–1.60	0.298

Favorable outcome (mRS 0–2), mRS (modified Rankin scale), CTA-CS (Computed tomography angiography collateral score), good collaterals (CTA-CS 3), fair collaterals (CTA-CS 0–2), OR (odds ratio), AOR (adjusted odds ratio), CI (confidence interval)

### Infarct core volume distribution in relation to mortality

The distribution of infarct core volume values with respect to patient mortality was analyzed. The mean infarct core volumes significantly differed between deceased (*n* = 27) and surviving (*n* = 181) patients, with higher values observed in the deceased group (11.2 mL vs. 6.2 mL, *p* = 0.002).

## Discussion

Our study demonstrates that automatically assessed CC and infarct core are predictive of functional outcome in AIS patients treated with EVT.

We found that a higher CTA-CS category is associated with a lower mRS score and a favorable outcome at 90 days. Furthermore, patients with a favorable outcome had a higher proportion of good collaterals (68%) compared to the poor outcome group (53.3%). Our findings are consistent with conclusions from previous researches and meta-analyses, confirming the important role of CC in preserving the penumbra from advancing to infarct core [17–19]. Additionally, our univariate logistic regression model showed that CS is a predictive factor of a favorable outcome at 90 days (*p* = 0.014). However, contrary to our expectations, CS lost significance in the multivariable model after adjusting for infarct core. We believe this might be due to a small group of patients in the CTA-CS 0 and 1 categories, which could have influenced the statistical analysis. Perhaps enlarging the study population might result in a more representative distribution of patients among the CTA-CS categories. In a recent study, the multivariable logistic regression yielded a result similar to ours, as automated CS did not emerge as a significant predictor of good functional outcomes [20]. Conversely, in the IMS III trial, multivariable analyses demonstrated that a robust collateral grade significantly predicted a good clinical outcome (mRS score ≤ 2) at 90 days [21]. In this study, CC was evaluated through visual scoring using a 5-point scale, which could serve as a distinguishing factor between our study and the IMS III trial, potentially elucidating the disparities in CS’s predictive value for functional outcomes.

It is noteworthy that not all studies have affirmed the significance of CC. For instance, in a prespecified analysis of the DEFUSE 3 trial, the presence of good collaterals did not demonstrate an association with achieving functional independence, defined as an mRS score of ≤ 2 [22]. The trial focused on patients within the late therapeutic window (> 6 h after stroke onset), introducing bias by forming a study group not representative of the general population. Additionally, CC evaluation relied on visual scoring with two different scales, which were subsequently dichotomized into only two collateral categories. These factors might account for the observed variations in results compared to our findings.

Turning to the impact of infarct core volume on patient outcomes, our study reveals significant associations. Among patients achieving favorable outcomes, the mean infarct core volume was lower compared to the poor outcome group (5 mL vs. 8.6 mL, *p* = 0.003). Additionally, when comparing surviving and deceased patients, the mean infarct core volume was significantly higher in the deceased group (11.2 mL vs. 6.2 mL, *p* = 0.002). The univariate and multivariable logistic regression models reinforced our findings, showing an inverse association between infarct core and favorable outcomes at 90 days. These observations align with the concept that patients with smaller infarct cores tend to have more favorable outcomes [11, 12]. Moreover, prior reports have noted a cutoff value of 70–100 mL for infarct core volumes, beyond which patients are significantly more prone to poor clinical outcomes despite successful reperfusion [23, 24].

The main purpose of our multivariable logistic regression model was to evaluate the combined predictive value of CC and infarct core for favorable outcomes. Our two models incorporated infarct core volume and both alternatives of CS: CTA-CS and dichotomized collaterals (good vs. fair). As mentioned earlier, only infarct core emerged as an independent predictor, suggesting that the predictive value lies primarily with the infarct core. Stated another way, our findings support the idea that the size of the infarct core is more relevant than CC in predicting functional outcomes in AIS patients treated with EVT. In alignment with our findings, a recent study by Tan et al. found that infarct core volume outperformed CC in predicting functional outcomes when their combined value was evaluated [25]. Indeed, infarct core volume effectively predicted both excellent and poor outcomes, whereas CC showed no significant association. Specifically, an increase in infarct core volume by 1 mL was associated with a lower likelihood of achieving an excellent outcome (mRS score 0–1, OR 0.942), which is very similar to our results (AORs 0.95 and 0.96). However, it is important to note several distinctions between our studies. Firstly, they utilized a different method for CC assessment, employing visual scoring on a 6-point scale based on multiphase CTA (mCTA). Secondly, their definition of functional outcome differed from ours, comprising excellent outcomes defined as mRS score 0–1 and poor outcomes as mRS score 5–6. Despite variations in methodology and outcome definitions, our findings exhibit reasonable agreement. Building on these insights, Fanou et al. investigated similar themes in their study, evaluating the combined predictive value of CC and infarct core volume in predicting mRS score > 2 outcome in patients with confirmed anterior circulation stroke treated with IVT within 4.5 h of symptom onset [14]. Their modeling was conducted on subgroups of patients, divided into recanalized and non-recanalized groups, whereas our modeling encompassed the entire population without further subdivision, highlighting a significant distinction. In their non-recanalized group, infarct core volume independently predicted the outcome, whereas in the recanalized group, it was marginally significant. However, CC did not reach significance in either subgroup. These observations are therefore consistent with our findings, underscoring the significant role of infarct core and noting the lack of significance for CC, despite the differing treatment approaches employed in each study. Of note, their study focused solely on IVT therapy, whereas our patients were treated with EVT. Furthermore, as in the previously discussed research, this study also employed a visual scoring method for CC assessment. Although our studies differ in treatment approach, methodology, and subgroup analysis, this highlights the robustness of the finding regarding the importance of infarct core in influencing stroke outcomes.

Complementing our insights into the prognostic value of infarct core and CC, the adoption of automated methodologies has reshaped their evaluation in stroke care, widely recognized for their quantitative, reliable, and efficient assessment [20, 26–28]. While automated estimations of infarct core volumes have been integrated into AIS management guidelines, the evaluation of CC remains varied, employing methods ranging from visual scoring to automated assessment [26, 28, 29]. Automatically assessed CC and infarct core have been individually linked to patient outcomes in AIS in previous research; however, their combined predictive value remains unexplored [16, 26, 28]. Our study seeks to fill this gap by evaluating the combined predictive value of these variables on functional outcomes, leveraging AI-powered software for automatic assessment. This integrative approach represents a novel contribution to stroke research, distinct from studies such as those by Tan et al. and Fanou et al., which utilized expert-conducted visual scoring for CC evaluation. To our knowledge, this integrative approach has not been addressed in prior literature. By employing automated methods, we seek to enhance the reliability and reproducibility of our findings, offering insights into the potential of these assessments to guide clinical decisions in AIS patients more effectively.

Our research has several limitations. Firstly, we used a CT scanner with a moderate number of slices (40-slice scanner), which affects the speed of data acquisition, particularly important in acquiring perfusion data, thereby restricting the dataset size obtained within the specified time frame. Such a restricted dataset could result in erroneous and inaccurate calculations by the software during the post-processing analysis of perfusion parameters. Secondly, our study was not conducted in a blinded manner, meaning that the experts involved were aware of the patients’ conditions during assessments, potentially introducing bias. Lastly, the CS in our study was derived from a single evaluation, which may not fully encompass the dynamic nature of CC. This static measurement approach could lead to inaccurate or misleading data.

## Conclusion

Our study demonstrated that automatically assessed CC and infarct core are predictive of functional outcomes in AIS patients treated with EVT. Higher CS and lower infarct core volumes were associated with a favorable functional outcome at 90 days, defined as an mRS score of ≤ 2. In univariate analysis, both factors predicted favorable outcomes. However, in the multivariable model evaluating their combined effect, only infarct core remained significant, suggesting that it alone has a more substantial impact on predicting outcomes compared to CC. Further studies are needed to elucidate the lack of significance of automatically assessed CC when combined with infarct core.
