# Correction to: Automated assessment of collateral circulation and infarct core: predictors of functional outcomes in acute ischemic stroke following endovascular thrombectomy

**DOI:** 10.1007/s00234-025-03604-2

**Published:** 2025-04-30

**Authors:** Ingrid Požar, Fajko F. Bajrović, Lan Umek, Katarina Šurlan Popović

**Affiliations:** 1 Department of Radiology, Izola General Hospital, Izola, Slovenia; 2 Faculty of Medicine, University of Ljubljana, Ljubljana, Slovenia; 3 Department of Vascular Neurology and Intensive Care, University Medical Centre Ljubljana, Ljubljana, Slovenia; 4 Institute of Pathophysiology, Faculty of Medicine, University of Ljubljana, Ljubljana, Slovenia; 5 Faculty of Public Administration, University of Ljubljana, Ljubljana, Slovenia; 6 Institute of Radiology, University Medical Centre Ljubljana, Ljubljana, Slovenia

**Correction to: Neuroradiology**

10.1007/s00234-024-03519-4

The original version of this article unfortunately contained a mistake.

The below text found in the body should be capture as Table 1 footnote;

Missing data for *1, **5, ***13, and ****21 patients.

CTA-CS (Computed tomography angiography collateral score: 0, < 10% of the affected middle cerebral artery territory filled by collateral supply compared to the contralateral unaffected hemisphere; 1, 10–50%; 2, 51–90%; 3, > 90%. There were no patients in the CTA-CS 0 category), IVT (intravenous thrombolysis), ASPECTS (Alberta stroke program early CT score), mRS (modified Rankin scale), mTICI (modified thrombolysis in cerebral infarction), time to CT (time from stoke onset or last known well to baseline CT), ICA (internal carotid artery), MCA (middle cerebral artery), ACA (anterior cerebral artery). Statistically significant *p*-values are italicized.

The below text found in the body should be capture as Table 2 footnote;

Missing data for *1, **5, ***13, ****21, and *****23 patients.

mRS (modified Rankin scale), favorable outcome (mRS 0–2), poor outcome (mRS 3–6), IVT (intravenous thrombolysis), ASPECTS (Alberta stroke program early CT score), CTA-CS (Computed tomography angiography collateral score: 0, < 10% of the affected middle cerebral artery territory filled by collateral supply compared to the contralateral unaffected hemisphere; 1, 10–50%; 2, 51–90%; 3, > 90%), good collaterals (CTA-CS 3), fair collaterals (CTA-CS 0–2), mTICI (modified thrombolysis in cerebral infarction), successful EVT (mTICI 2b-3), unsuccessful EVT (mTICI 0-2a), time to CT (time from stoke onset or last known well to baseline CT), time to EVT (time from stoke onset or last known well to endovascular thrombectomy), ICA (internal carotid artery), MCA (middle cerebral artery), ACA (anterior cerebral artery). Statistically significant *p*-values are italicized.

The below text found in the body should be capture as Table 3 footnote;

Favorable outcome (mRS 0–2), mRS (modified Rankin scale), CTA-CS (Computed tomography angiography collateral score), good collaterals (CTA-CS 3), fair collaterals (CTA-CS 0–2), OR (odds ratio), AOR (adjusted odds ratio), CI (confidence interval).

In Table 3 entries, the two occurrences of ‘p’ in p-value should be italicize.

The original article has been corrected.

